# Validation of Polar OH1 optical heart rate sensor for moderate and high intensity physical activities

**DOI:** 10.1371/journal.pone.0217288

**Published:** 2019-05-23

**Authors:** Imali T. Hettiarachchi, Samer Hanoun, Darius Nahavandi, Saeid Nahavandi

**Affiliations:** Institute for Intelligent Systems Research and Innovation, Deakin University, Waurn Ponds, VIC 3216, Australia; James Cook University College of Healthcare Sciences, BRAZIL

## Abstract

**Background:**

Optical measurement techniques and recent advances in wearable technology have made heart rate (HR) sensing simpler and more affordable.

**Objectives:**

The Polar OH1 is an arm worn optical heart rate monitor. The objectives of this study are two-fold; 1) to validate the OH1 optical HR sensor with the gold standard of HR measurement, electrocardiography (ECG), over a range of moderate to high intensity physical activities, 2) to validate wearing the OH1 at the temple as an alternative location to its recommended wearing location around the forearm and upper arm.

**Methods:**

Twenty-four individuals participated in a physical exercise protocol, by walking on a treadmill and riding a stationary spin bike at different speeds while the criterion measure, ECG and Polar OH1 HR were recorded simultaneously at three different body locations; forearm, upper arm and the temple. Time synchronised HR data points were compared using Bland-Altman analyses and intraclass correlation.

**Results:**

The intraclass correlation between the ECG and Polar OH1, for the aggregated data, was 0.99 and the estimated mean bias ranged 0.27–0.33 bpm for the sensor locations. The three sensors exhibited a 95% limit of agreement (LoA: forearm 5.22, -4.68 bpm; upper arm 5.15, -4.49; temple 5.22, -4.66). The mean of the ECG HR for the aggregated data was 112.15 ± 24.52 bpm. The intraclass correlation of HR values below and above this mean were 0.98 and 0.99 respectively. The reported mean bias ranged 0.38–0.47 bpm (95% LoA: forearm 6.14, -5.38 bpm; upper arm 6.07, -5.13 bpm; temple 6.09, -5.31 bpm), and 0.15–0.16 bpm (95% LoA: forearm 3.99, -3.69 bpm; upper arm 3.90, -3.58 bpm; temple 4.06, -3.76 bpm) respectively. During different exercise intensities, the intraclass correlation ranged 0.95–0.99 for the three sensor locations. During the entire protocol, the estimated mean bias was in the range -0.15–0.55 bpm, 0.01–0.53 bpm and -0.37–0.48 bpm, for the forearm, upper arm and temple locations respectively. The corresponding upper limits of 95% LoA were 3.22–7.03 bpm, 3.25–6.82 bpm and 3.18–7.04 bpm while the lower limits of 95% LoA were -6.36–(-2.35) bpm, -6.46–(-2.30) bpm and -7.42–(-2.41) bpm.

**Conclusion:**

Polar OH1 demonstrates high level of agreement with the criterion measure ECG HR, thus can be used as a valid measure of HR in lab and field settings during moderate and high intensity physical activities.

## Introduction

HR is one of the key metrics that can show and track human physical activity levels. HR is modulated not only by physical activities but also by affective and cognitive states including cognitive load, stress, anxiety, fatigue and many other factors such as sleep, nutrition, illness, meditation and caffeine intake. It is considered a vital physiological measure that can be relied upon for assessing human performance in real-life scenarios across different application domains such as health and wellbeing, emergency services, sports and training [1, 2].

Fitness tracking devices that support HR measurements are broadly classified into wrist-worn (e.g. Fitbit Charge, Apple watch, Basis Peak and Polar RS800CX), arm worn (e.g. Scosche Rhythm+, Polar OH1 and Wahoo Tickr Fit), temple-worn (e.g. Moov HR Sweat) and chest-worn (e.g. Polar H10, Garmin HRM and Wahoo Fitness Tickr X) devices. Though a plethora of HR monitors exists, it is worth noting that when considering them for real-life or out-of-laboratory scenarios, ease of wearability, less cumbersomeness, comfort and data accessibility are among many factors that influence their user acceptance. Another vital factor is the accuracy of HR measurements, as inaccurate data measurements affect their ability to deduce robust conclusions for many applications.

HR chest strap devices rely on electrocardiac sensors for their technology to detect and monitor HR. They have demonstrated high agreement to the gold standard, ECG [3, 4]; however, they have their own limitations. The electrodes of the chest straps require to be hydrated to ensure good conductivity. Further, they need to be strapped up under garments, which can be cumbersome often causing discomfort especially with increased sweating during high intensity physical activities. These factors supported the interest towards easily worn and conveniently smaller, wrist, arm and temple worn HR monitors. However, the downside of these monitors is their compromised accuracy compared to chest strap monitors.

On the other hand, wrist, arm and temple worn monitors rely on the photoplethysmography (PPG) technology to monitor HR. Although, PPG is a simple, reliable and low-cost optical measurement technique, the accuracy of PPG activity monitors are affected by artifacts associated with the sensor movement [5–7], and thus there is a need for rigorous validation against the criterion measure ECG.

Recent studies investigated the accuracy of Polar OH1 during yoga sequences [8] and during a 7-difficulty level working memory cognitive load task [2]. The yoga protocol [8] was categorised as a light to moderate physical activity and the criterion measure was based on the Polar H7 chest strap. In contrast to the aforementioned studies, the objective of this study is to assess and validate the accuracy of the Polar OH1 arm worn HR monitor under different moderate to high intensity physical activities. We investigated the validity of the Polar OH1 by assessing the agreement with criterion measure ECG under a exercise protocol consisting of treadmill and spin bike. Further, we extended the Polar OH1 accuracy tests of our study to include a third wearing location for the sensor; the temple, in addition to its recommended wearing positions forearm and upper arm. This validation on the temple is motivated based on recommendations for a similar HR monitor, the Moov HR Sweat.

## Materials and methods

### Participants

Twenty-four healthy right handed participants (12 males and 12 females) in the age group 21-38 years (age = 28.09 ± 5.50 years, weight = 66.57 ± 14.65 kg, height = 170.72 ± 9.98 cm) volunteered to participate in this study. Table 1 shows the mean, standard deviation (SD) and range for age, height, weight, and body mass index (BMI) for the participants, grouped based on their gender. Ethics approval for data collection was granted by the Human Ethics Advisory Group (HEAG) of the Faculty of Science Engineering and Built Environment, Deakin University, Australia. A pre-screening questionnaire was used to assess whether the participants were physically able to perform the exercise protocol without health complications and minimal risk of injury. The pre-screening questionnaire is provided in the supplementary material section. Participants free of any cardiovascular or respiratory diseases and physically fit were considered for the experiment. Participants were fully briefed about the study and their written consent was acquired for data collection before the start of the experiment.

**Table 1:** Participants’ characteristics. BMI: body mass index.

Demographics	Males		Females	
	Mean (SD)	Range	Mean (SD)	Range
Age (years)	26 (4.8)	21–33	30 (5.48)	21–38
Height (cm)	176.8(5.77)	168–187	164.09(9.47)	150–175
Weight (kg)	76.33(10.29)	60–97	55.91(10.77)	44–76
BMI	24.43(3.26)	20.02–30.68	20.86(4.57)	16.33–33.33

### Experimental design

Initially, all participants had their body weight and height measured. Subsequently, participants were prepared for ECG data collection and the three Polar OH1 sensors were placed on their forearm, upper arm and temple for HR collection. The ECG and HR measurements from each of the Polar OH1 sensors were recorded on the same computer, where the Polar OH1 HR measurements were time stamped using the system clock.

**Fig 1:**
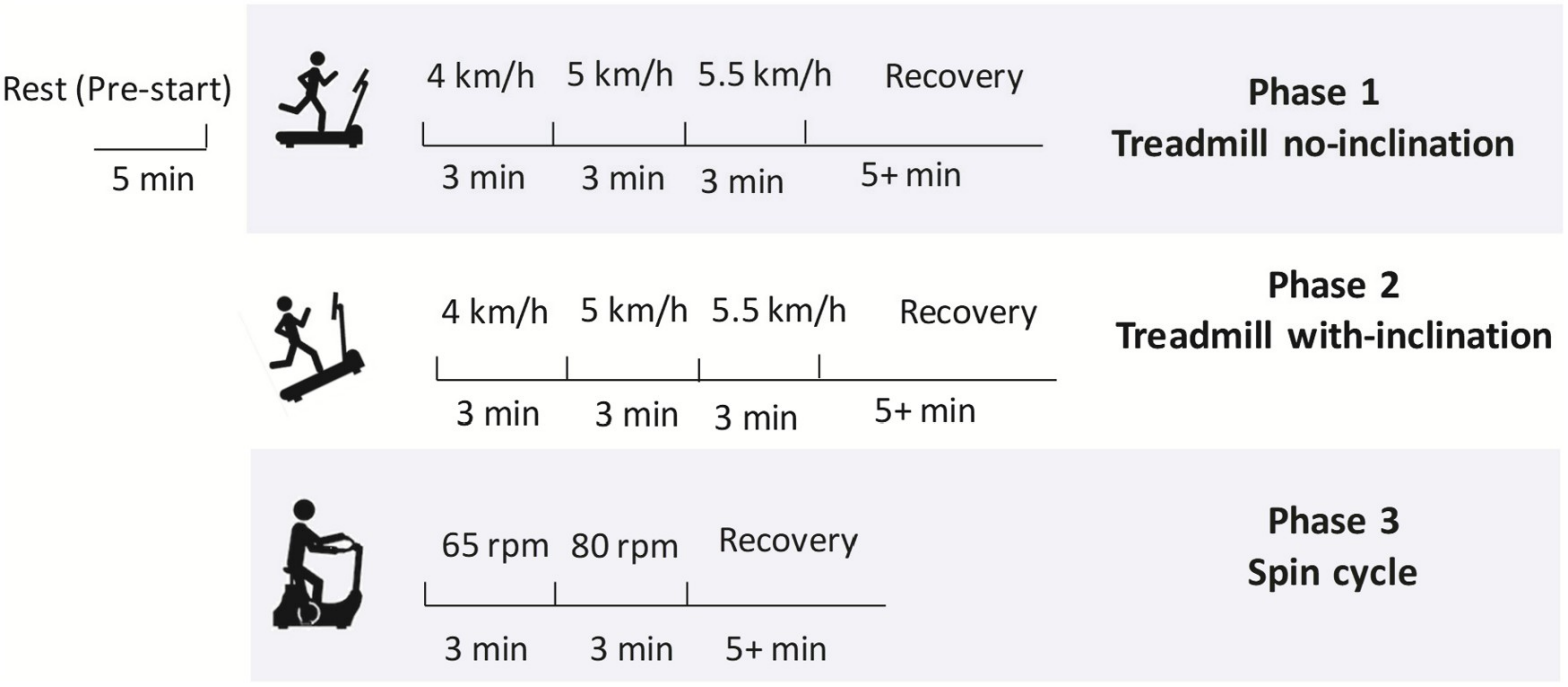
Experimental protocol. Total duration is 44–60 minutes due to variable recovery times.

During data acquisition each participant completed a 44–60 minutes test protocol as shown in Fig 1. The protocol was established using a pilot study and the details are provided in the supplementary material section. The test consisted of three phases, each phase are separated by a recovery period. The three phases were treadmill walking with no inclination (low-moderate exertion), treadmill walking with inclination (moderate-high exertion) and spin bike exercise (moderate-high exertion) respectively. A BodyWorx Boston M1 treadmill and BodyWorx A117 Spin Bike was used to perform the protocol. Each of phase one and two on the treadmill lasted for 9 minutes, while the spin bike phase lasted for 6 minutes. During the treadmill activity, three different speed levels of 4, 5 and 5.5 km/h were used. Participants were instructed to maintain each speed for 3 minutes. Participants observed a timer on the treadmill display to help them know when it is the time to change their speed, while a research team member closely monitored the timer and the speeds during change process. A manual marker was set on the ECG recordings to mark the time when the change of speed has occurred.

During phase two, the inclination of the treadmill was increased to its maximum angle of 6.1° and the three speed levels were each walked for 3 minutes. The spin bike phase of the experiment was divided into two episodes, in which the participants were asked to cycle for 3 minutes at 60 (±5) revolutions per minute (rpm) and another 3 minutes at 80 (±5) rpm. Participants were initially seated resting for 5 minutes before starting the experiment’s first phase. At the end of each phase, participants were allowed a minimum recovery time of 5 minutes; however, they were allowed a longer resting period if they required. Sufficient time was allowed to recover before switching between phases to minimise exhaustion. For data analysis, the centre 3 minutes of the 5 minutes resting recording were only used and the first 3 minutes of the recovery were only considered. In each phase, participants’ speed, style of walking and cycling were noted. Observations included, walking with hands free in air, walking with holding the treadmill rails for support, cycling seated or non-seated and cycling while holding or not holding the bike handle bar.

### Criterion measure: Electrocardiography (ECG)

**Fig 2:**
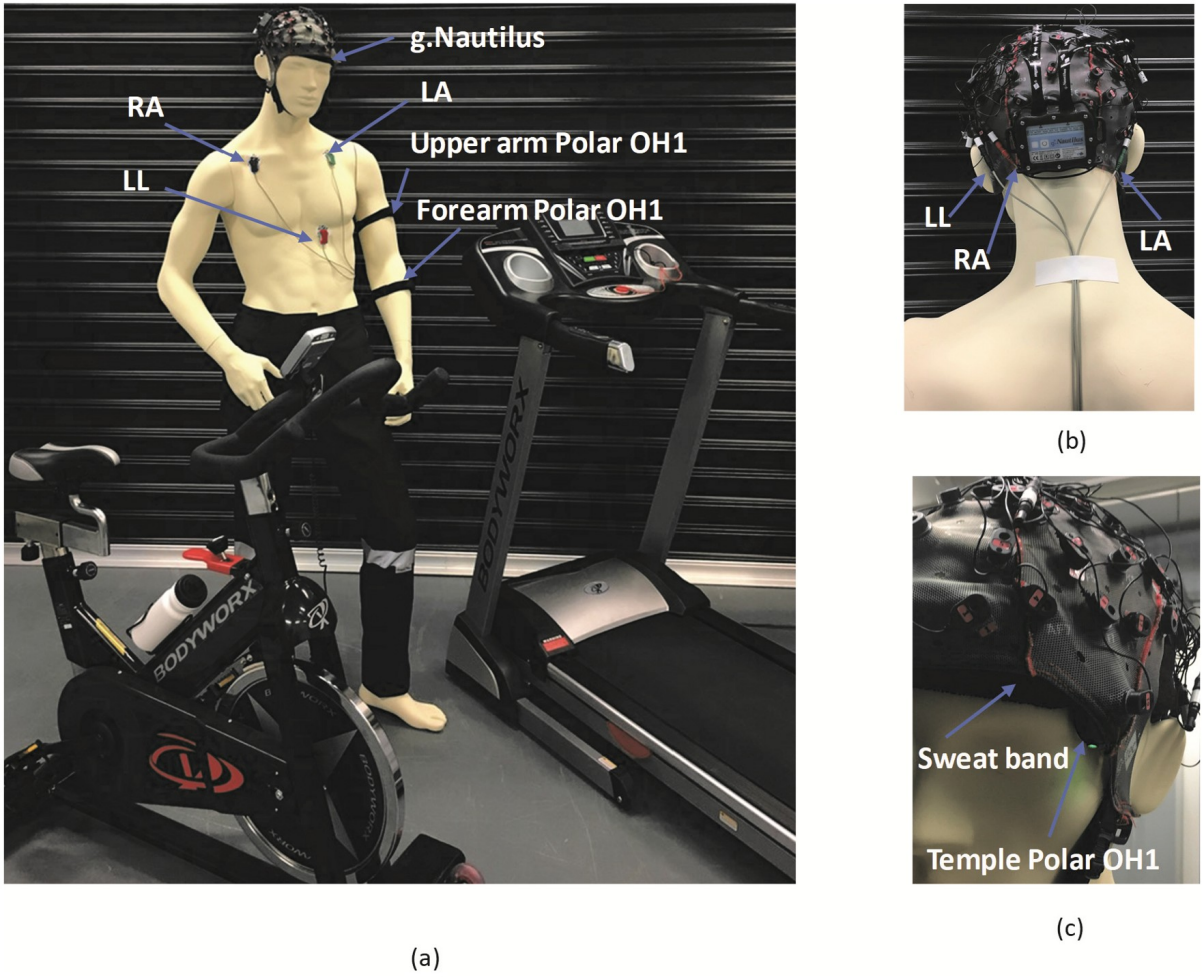
Participant preparation. (a) ECG leads and Polar OH1 arm sensor placement on the participant. (b) The ECG leads connected to the g.Nautilus device. RA and LL are connected to two detachable electrodes, while LA is connected to the reference (*REF*) channel of the g.Nautilus. (c) The Polar OH1 sensor on the temple is secured by a sweatband. RA: Right arm electrode, LA: Left arm electrode, LL: Lower left chest electrode.

ECG was recorded via a 64-channel wireless g.Nautilus active electrode multipurpose biosignal acquisition system (g.tec medical engineering GmbH, Austria). The cap-type g.Nautilus was worn by the participants. Three electrodes were attached to the participant’s upper torso as shown in Fig 2(a), during data collection. Skin preparation at the electrode placement sites was performed, by cleansing with alcohol wipes and light abrasion and shaving to minimise noise artifacts and in order to improve the signal quality. Silver/silver-chloride self-adhesive electrodes were placed on the participant’s upper torso, under the right clavicle bone (RA), left clavicle bone (LA) and the lower left chest (LL) regions. The connection leads were connected to the g.Nautilus cap electrodes as shown in Fig 2(b).

To minimise artifacts due to electrode movement, the ECG wires were secured to the participant’s body using adhesive medical tape. The voltage difference between electrode sites, LL and RA (i.e., LL-RA) were recorded as 1-lead ECG with a sampling rate of 250Hz. A 0.1-100Hz bandpass filter and a 50Hz notch filter was used as internal filter parameters during ECG acquisition.

#### ECG derived heart rate

The raw ECG recordings were pre-processed and analysed using MATLAB scripts, to generate a time synchronous ECG-based criterion HR. First the portions of the ECG recordings with extremely noisy signals were manually marked and excluded. Subsequently, the QRS complexes of the ECG signals were detected using the Pan-Tompkins QRS detection algorithm [9]. Then the R-peak series (tachogram) was obtained by calculating the intervals between successive R peaks (RR interval). The R-peak series is then examined and corrected for any missed and/or extra beats using a quotient filter [10, 11].

**Fig 3:**
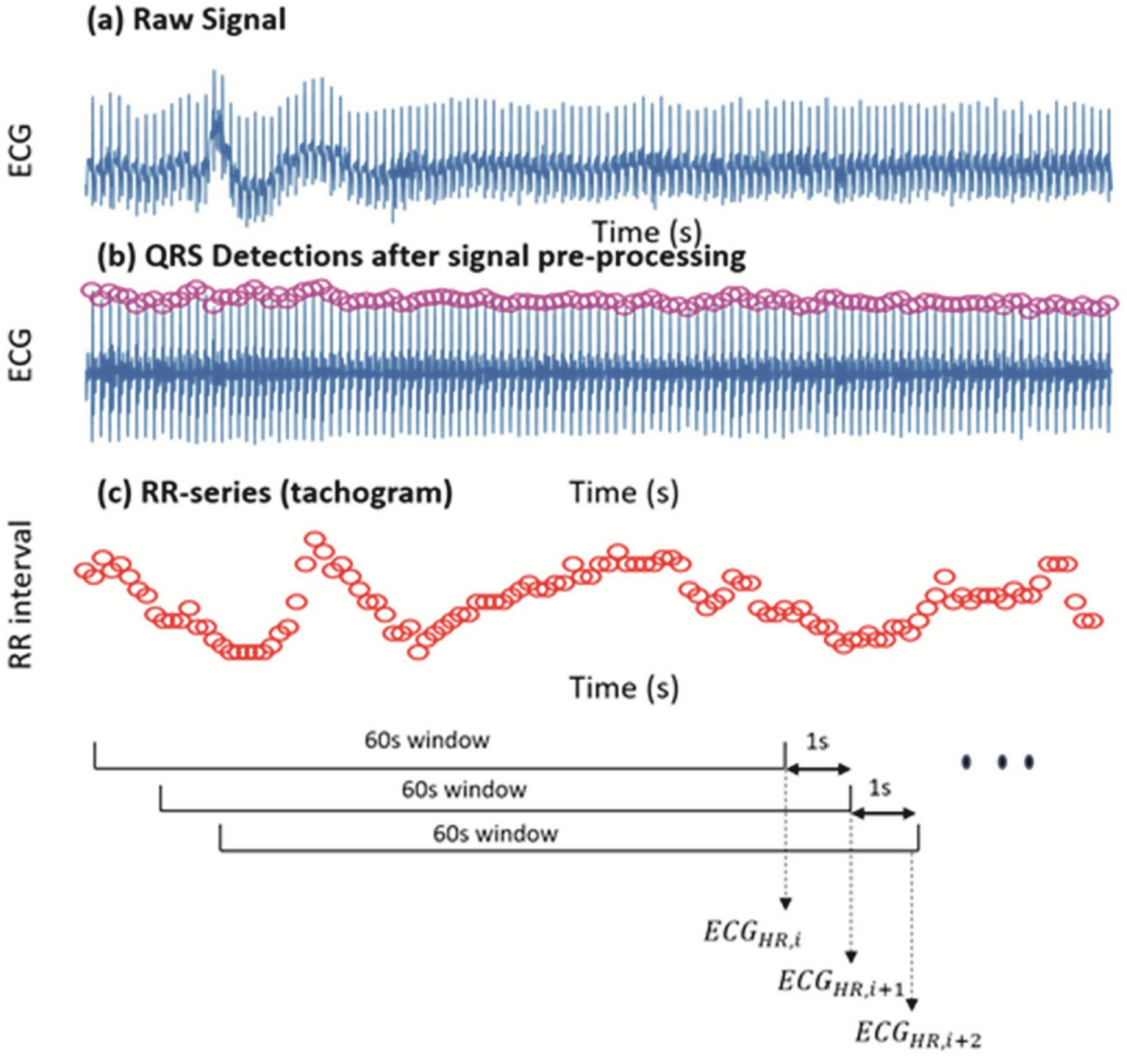
Pipeline of deriving the criterion HR from ECG. *ECG*_*HR*,*i*_ denotes the HR derived at the *i*^*th*^ iteration.

Subsequently, HR was derived by counting the number of R-peaks during a 60s (15000 samples) period, and this is reported in bpm against the end-time of each window. A moving window approach was used to derive the criterion HR every second, to obtain a HR series at the same sampling frequency of the Polar OH1, which read the HR every second. At each iteration the 60s window was moved by 1s (250 samples) to derive the criterion HR from ECG (*ECG*_*HR*_) (Fig 3).

### Polar OH1 setup

Polar recommends OH1 optical HR monitor to be worn on the left or right forearm or upper arm. During this study, both recommended locations were considered with an additional location on either the left or right temple. The temple electrode was placed under the g.Nautilus cap and secured with a sweatband (headband) worn under the cap. Fig 2(c) shows the Polar OH1 temple placement. None of the participants reported discomfort or movement of the sensor. Half of the participants wore the sensors on the dominant arm (right arm) and the other half of the participants wore the sensors on the non-dominant arm (left arm). The Polar OH1 on the temple was placed on the same side of the body as the arm worn sensors. To eliminate any particular confounding bias of the three Polar sensors, they were randomly selected for each sensor position. That is, no particular Polar OH1 sensor was assigned to any specific location (i.e., upper arm, forearm or temple) throughout the study. The three Polar OH1 sensors were assigned random sensor locations for every participants. However, the random selection was counterbalanced to ensure each OH1 had a probability of 1/3 for getting assigned to the three sensor sites (upper arm, forearm and temple). Hereafter, the Polar OH1 HR readings of the forearm, upper arm and temple are referred to as *PL*_*FA*_, *PL*_*UA*_ and *PL*_*TM*_ respectively.

A custom data logger was developed to interface simultaneously to the three Polar OH1 sensors utilizing Bluetooth Low Energy (BLE) technology. The logger software exported the time stamped HR measurements of the three Polar sensors to a CSV comma separated file for off-line processing.

## Statistical analysis

The derived *ECG*_*HR*_ and exported *PL*_*FA*_, *PL*_*UA*_ and *PL*_*TM*_ data were imported into MATLAB for pre-processing and analysis. At some instances, the Polar OH1 data measurements were missing due to low skin contact or loss in Bluetooth connection. On average about 5% of the data was lost from the Polar measurements. Therefore, time synchronisation of the *ECG*_*HR*_ and the Polar OH1 sensor recordings was achieved by considering only the times where all data measurements were available.

Intraclass correlation coefficient (ICC) [12, 13] and Bland-Altman analysis [14, 15], taking into account the repeated measures was used in the study to analyse the agreement between the Polar OH1 measured HR and the *ECG*_*HR*_. ICC reflects not only the degree of correlation but also agreement between measurements. ICCs were calculated using a two-way mixed effects model assessing for absolute agreement between 1 second *ECG*_*HR*_ and 1 second Polar OH1 measurements. In order to estimate any systematic bias, graphical Bland-Altman plots were constructed by plotting the difference of the *ECG*_*HR*_ and time-synced Polar OH1 readings against the mean of the two measurements. The mean bias of the agreement between the two measurements equalled the mean difference. Further the 95% LoA were calculated using the formula,

LoA=Meanbias±1.96×standarddeviationofthedifference. (1)

where, the standard deviation of the differences is calculated using the total variance for single differences on different subjects, estimated by the sum of two components. The two components are the variance of multiple differences of the between method HR estimated using one-way analysis of variance (ANOVA) for the same subject (within subject variance) and for differences between the average difference across subjects (variance representing heterogeneity) [15].

This was repeated for the three sensor locations, stratified by high and low HR zones with respect to the mean ECG HR and stratified by different phases of the experiment.

To determine whether there are any statistically significant differences between the Polar HR measurements at the three sensor locations, a non-parametric Kruskal-Wallis test was conducted for the three Polar OH1 sensor locations (*PL*_*FA*_, *PL*_*UA*_ and *PL*_*TM*_) HR values at the same time. Kruskal-Wallis is an alternative test to the one-way analysis of variance (ANOVA), as normality of the sample is not guaranteed.

## Results

### Stratified heart rate data

The first analysis carried out was based on aggregated data for each of the sensor locations. Both the treadmill and spin bike exercise sessions with varying physical load and rest/recovery intervals were aggregated in the analysis, which produced *n* = 25858 data points. Table 2, presents the descriptive statistics, including the mean and standard deviation (SD) of the *ECG*_*HR*_ and three Polar OH1 sensors.

**Table 2:** Descriptive statistics of criterion measure and the three Polar sensor measurements.

	Minimum HR (bpm)	Maximum HR (bmp)	Mean ± SD (bpm)
*ECG*_*HR*_	54	178	112.15 ± 24.52
*PL*_*FA*_	52	180	112.42 ± 24.52
*PL*_*UA*_	53	180	112.47 ± 24.47
*PL*_*TM*_	52	181	112.43 ± 24.50

**Fig 4:**
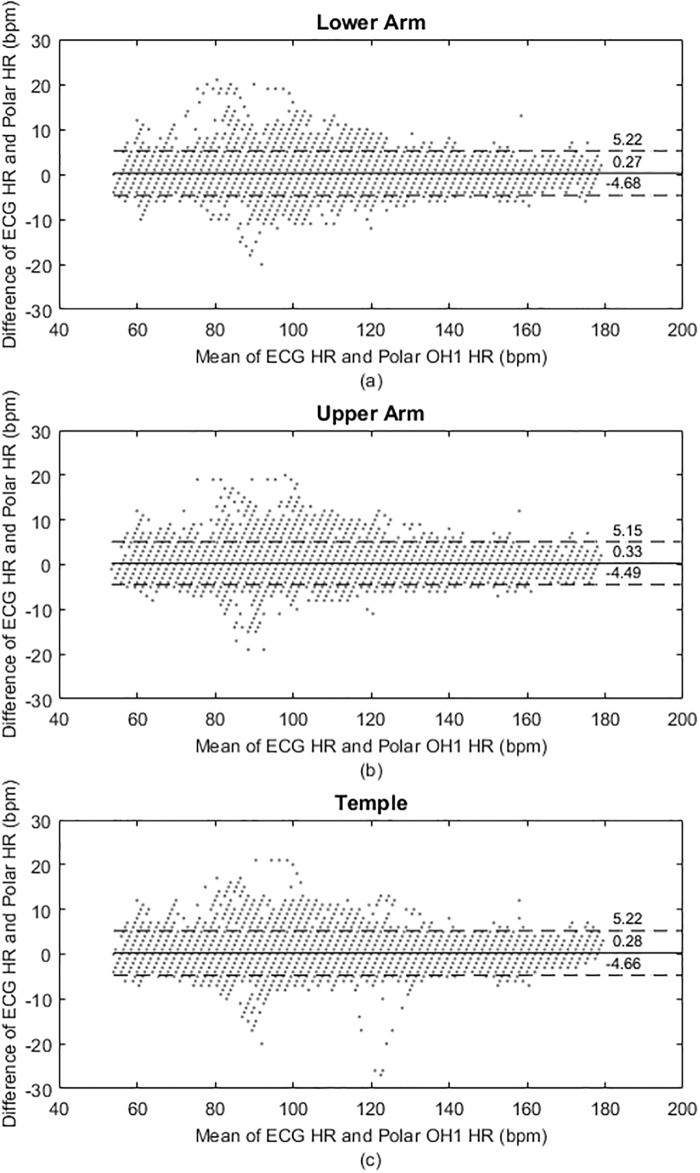
Aggregate data analysis results. Bland-Altman plots indicating mean bias scores and 95% limits of agreement (LoA) for the three sensor locations; (a) forearm (b) upper arm and (c) temple.

Fig 4 shows, the Bland-Altman plots for the time-synced *ECG*_*HR*_ and Polar HR data that were aggregated, while Table 3 summarises the statistical analysis results.

**Table 3:** Results for the aggregated data at different Polar sensor locations (*n* = 25858), *p* < 0.0001*.

	*ICC* (95% CI)	Mean bias (bpm)	95% upper LoA (bpm)	95% lower LoA (bpm)
*PL*_*FA*_	0.9946* (0.9944, 0.9949)	0.27 ± 2.52	5.22	-4.68
*PL*_*UA*_	0.9949* (0.9946, 0.9951)	0.33 ± 2.46	5.15	-4.49
*PL*_*TM*_	0.9947* (0.9944, 0.9949)	0.28 ± 2.52	5.22	-4.66

CI: confidence interval

### Intensity of physical activity

Next, the performance of the Polar sensors in different ranges of HR; i.e. high HR zone vs low HR zone, was analysed using a cut-off value based on the mean of the criterion measure HR (*ECG*_*HR*_) [16]. The mean of *ECG*_*HR*_ is 112.15 ± 24.52 bpm (Table 2). Therefore, the HR values of aggregated data were considered in ranges with respect to the mean ECG derived HR (Table 4).

**Table 4:** Data for different HR ranges with respect to the mean HR derived from ECG.

	Data for *ECG*_*HR*_ < 112 bpm (*n* = 13675)			Data for *ECG*_*HR*_ > 113 bpm (n = 12183)		
	*PL*_*FA*_	*PL*_*UA*_	*PL*_*TM*_	*PL*_*FA*_	*PL*_*UA*_	*PL*_*TM*_
Mean(SD) (bpm)	94.01 (13.80)	94.10 (13.73)	94.02 (13.71)	133.08 (15.97)	133.09 (15.98)	133.08 (16.00)
ICC (95% CI)	0.9767 (0.9753,0.9779)	0.9776 (0.9758,0.9793)	0.9770 (0.9756,0.9783)	0.9924 (0.9921,0.9927)	0.9928 (0.9925,0.9931)	0.9921 (0.9918,0.9924)
Mean bias (bpm)	0.38 ± 2.94	0.47 ± 2.86	0.39 ± 2.91	0.15 ± 1.96	0.16 ± 1.91	0.15 ± 1.99
95% upper LoA (bpm)	6.14	6.07	6.09	3.99	3.90	4.06
95% lower LoA (bpm)	-5.38	-5.13	-5.31	-3.69	-3.58	-3.76

### Type of exercise

Table 5, presents the descriptive statistics and validation metrics for each sensor for the entire protocol. It can be noted that throughout the entire protocol the highest and lowest correlation values were reported during the 80 rpm cycling and rest phases respectively. All activities and intensities report a strong ICC value > 0.9.

**Table 5:** Descriptive statistics and the validation metrics for the entire protocol. All data other than *ICC* given in bpm.

	*n*	Rest	Treadmill no inclination			Treadmill inclination			Cycle		Recovery
			4 km/h	5 km/h	5.5 km/h	4 km/h	5 km/h	5.5 km/h	60 rpm	80 rpm	
		2712	1439	1405	1311	1846	1944	2136	3192	3231	6642
Forearm	Mean(SD)	75.44 (9.94)	94.72 (9.08)	100.02 (8.28)	109.53 (10.94)	113.67 (9.55)	130.47 (9.25)	146.40 (12.50)	111.17 (13.95)	125.50 (17.81)	112.23 (26.17)
	Range (min–max)	52–102	73–116	84–119	91–139	81–135	103–152	120–172	81–160	89–179	57–180
	ICC	0.9480	0.9687	0.9652	0.9853	0.9620	0.9816	0.9933	0.9898	0.9949	0.9920
	ICC 95% CI upper bound	0.9516	0.9718	0.9686	0.9874	0.9652	0.9838	0.9947	0.9905	0.9955	0.9927
	ICC 95% CI lower bound	0.9440	0.9654	0.9614	0.9828	0.9584	0.9789	0.9912	0.9890	0.9942	0.9913
	Mean bias (SD)	-0.02 (3.23)	-0.08 (2.94)	-0.15 (2.45)	0.37 (2.02)	-0.01 (2.66)	0.34 (1.77)	0.43 (1.42)	0.20 (2.00)	0.34 (1.78)	0.55 (3.31)
	95% upper LoA	6.31	5.69	4.65	4.32	5.21	3.81	3.22	4.13	3.82	7.03
	95% lower LoA	-6.36	-5.84	-4.95	-3.58	-5.23	-3.13	-2.35	-3.72	-3.14	-5.93
Upper arm	Mean(SD)	75.71 (9.84)	94.85 (9.10)	100.18 (8.37)	109.58 (10.97)	113.81 (9.69)	130.44 (9.23)	146.39 (12.46)	111.14 (13.85)	125.55 (17.76)	112.21 (26.22)
	Range (min–max)	53–108	72–115	84–119	91–139	80–136	104–152	120–172	81–160	95–179	57–180
	ICC	0.9493	0.9656	0.9682	0.9842	0.9637	0.9828	0.9932	0.9912	0.9951	0.9925
	95% CI upper bound	0.9453	0.9619	0.9648	0.9812	0.9603	0.9805	0.9911	0.9905	0.9943	0.9918
	95% CI lower bound	0.9529	0.9689	0.9713	0.9866	0.9668	0.9848	0.9946	0.9918	0.9957	0.9931
	Mean bias (SD)	0.25 (3.15)	0.05 (3.33)	0.01 (2.32)	0.42 (2.09)	0.13 (2.60)	0.31 (1.71)	0.43 (1.44)	0.18 (1.86)	0.39 (1.73)	0.53 (3.21)
	95% upper LoA	6.43	6.57	4.57	4.52	5.22	3.65	3.25	3.82	3.77	6.82
	95% lower LoA	-5.93	-6.46	-4.54	-3.68	-4.97	-3.04	-2.38	-3.46	-2.30	-5.76
Temple	Mean(SD)	75.55 (9.56)	94.43 (9.15)	100.10 (8.33)	109.39 (10.92)	114.12 (9.44)	130.41 (9.22)	146.35 (12.54)	111.21 (13.88)	125.53 (17.77)	112.15 (26.22)
	Range (min–max)	52–101	72–113	84–119	91–139	81–136	105–152	120–172	81–161	95–179	57–181
	ICC	0.9459	0.9611	0.9663	0.9860	0.9654	0.9821	0.9934	0.9906	0.9954	0.9919
	95% CI upper bound	0.9418	0.9564	0.9627	0.9843	0.9609	0.9800	0.9917	0.9898	0.9946	0.9912
	95% CI lower bound	0.9497	0.9653	0.9696	0.9875	0.9693	0.9840	0.9946	0.9914	0.9960	0.9924
	Mean bias (SD)	0.08 (3.22)	-0.37 (3.60)	-0.08 (2.41)	0.23 (2.01)	0.43 (2.47)	0.28 (1.75)	0.38 (1.43)	0.24 (1.92)	0.37 (1.67)	0.48 (3.35)
	95% upper LoA	6.40	6.68	4.65	4.18	5.28	3.70	3.18	4.00	3.64	7.04
	95% lower LoA	-6.23	-7.42	-4.80	-3.72	-4.41	-3.14	-2.41	-3.51	-2.89	-6.09

### Effect of Polar sensor location

A One-sample Kolmogorov-Smirnov test indicated that the HR values recorded in *PL*_*FA*_ did not follow a normal distribution, *D*(25857) = 0.0391, *p* = 0(< 0.05). Similar results were found with *PLUA* (*D*(25857) = 0.0420, *p* = 0) and *PL*_*TM*_ (*D*(25857) = 0.0406, *p* = 0). The Kruskal-Wallis test results showed that there was no statistically significant difference between HR measurements recorded at the three sensors locations *F*(2, 77571) = 0.07, *p* = 0.9639.

## Discussion

The objective of the current study was to investigate the validity of HR measurements from the commercially available arm worn Polar OH1 HR monitor, which measures HR using the PPG technique. The Polar OH1 was validated against the criterion measure ECG using a protocol which included, walking on a treadmill at different speeds and inclinations and spin cycle exercise that both covered moderate and high intensity physical activities.

The study indicates good criterion-related validity of the Polar OH1 compared with ECG during moderate and high intensity physical activities. The ICC is very high at all sensor locations and at each phase of the protocol (> 0.9). Alongside other validation studies [3, 16, 17] for HR monitors, which recommend a mean bias < 3 bpm to determine validity, the Polar OH1 is in good agreement to the criterion measure ECG. The Kruskal-Wallis test results show that the three sensor locations does not affect the HR readings. Thus, for different applications the temple might be used for the Polar OH1 as an alternative wearing location.

A recent study on Scoche Rhythm+ [18], reported a Lin’s concordance correlation coefficient, *r*_*c*_ = 0.92, 0.84, 0.93 for treadmill, bike and rest phases, respectively. In an earlier study [19], the mean absolute error (MAE) on aggregated data for walking, running and cycling were reported as 4.83, 10.48, 6.75 bpm respectively. Another study on Scoche Rhythm+ [20] during moderate to intense physical activities using a treadmill protocol, reported a mean bias 0.3 bpm with a rather wide 95% LoA -18.6–19.3 bpm. Compared to these studies on a competitor arm worn device Scoche Rhythm+, the OH1 reported a strong intraclass correlation, smaller mean bias and narrower LoA, with respect to the criterion measure ECG. Thus, it is worth noting that not all arm worn devices work with the same level of agreement. This might be due to differences in their design including number of LED lights used, sensor placement and signal processing algorithms used.

The Polar OH1 agreement analysis results reported on a yoga physical activity [8] showed a mean bias of –0.76 bpm (95% CI: –2.06–0.53 bpm), while the Bland-Altman analysis 95% LoAs were -3.83–5.35 bpm. Our earlier study on different cognitive load levels [2], reported a mean bias of 0.06 bpm and a 95% LoA -4.34–4.46 bpm. Compared to results from these studies involving static protocols [2, 8], the results of this study on a dynamic exercise protocol show comparable degree of agreement of the Polar OH1 to the criterion ECG (Table 5).

Participants were asked to use a free style of preference while using the treadmill and the spin bike. From their recorded style during the sessions, we noticed that majority of the participants (19 out of 24) used a free style of walking at low speed (4 km/h) during phase 1 and 2 (i.e., with hands moving on the side of the body). Also, we noted that all participants held the hand rail of the treadmill at high speeds (5 and 5.5 km/h) during phase 1 and for all speeds during phase 2 (treadmill with inclination). Thus, it is worth noting that during the data collection, Polar OH1 readings were less affected by movement related artifacts.

A closer look at the data from our study revealed that the agreement of the Polar OH1 with ECG varies with the intensity of physical activity (Table 4). At lower HR, a lower agreement is observed compared to the agreement of the higher HR zone. Increased physical activity will result in improved blood perfusion, resulting in an increase in agreement between the Polar OH1 and the criterion ECG. Other studies on arm-based HR monitors [19, 20] have provided evidence to support this fact showing a reduction in mean average percentage error with increased speed of activity.

Compared to the proven accuracy of HR chest straps, the arm worn Polar OH1 carries the advantage of being more comfortable and ease of wearing, especially for females. This is an attractive characteristic when it comes to applications such as human performance monitoring in areas including simulation-based training, Defence and firefighting. For instance, during a simulation-based training task, the trainees can be taught to wear the Polar OH1 by themselves, which may increase motivation, the feeling of control and safety. This added advantage and the proven accuracy would increase the user acceptability of the Polar OH1.

It is worth to note that during the experiments there were occasional loss of skin contact of the Polar OH1 despite the tight fit of the armbands and the head sweatband where, the Polar OH1 transmits a zero value for the HR measurement. To correct this, the armband or sweatband was immediately adjusted, and the build-up times were marked manually and excluded from the analysis. This scenario needed attention to ensure that the measurements are accurate for the study conclusions.

A limitation of our study is that we have not considered higher diversity among participants. Although we have accounted for gender, the study was conducted under laboratory settings involving young and healthy volunteers. Results may vary for different subsets of individuals, including elders and cardiac patients. Also, it has been found that skin tone can effect the accuracy of the optical HR monitors [16, 18, 21]. Future validation studies on Polar OH1 should incorporate parameters such as age, medical background and skin color of participants to further confirm the validity of Polar OH1.

## Conclusion

The findings of the study shows that the Polar OH1 HR is in high agreement to the criterion measure ECG HR under moderate to high intensity physical activities. Therefore, when accurate HR measurements are required, Polar OH1 can be used in place of ECG or chest strap HR monitors during moderate to high intensity physical activities.

## Supporting information

S1 Dataset The dataset generated and analysed for this study. (XLS)

S1 Supporting Information The pre-screening questionnaire and the procedure for establishing the experimental protocol. (DOCX)
