# Supplementary material for: Validation of Polar OH1 optical heart rate sensor for moderate and high intensity physical activities
Source: PLoS One. 2019 May 23;14(5):e0217288. doi: 10.1371/journal.pone.0217288 (PMC6532910; doi:10.1371/journal.pone.0217288)
Supplement: S1 Supporting Information — (DOCX) [file pone.0217288.s002.docx]

**Pre-screening questionnaire for physical fitness assessment**

A sample of the questions included in the participants pre-screening questionnaire are as follows:

- If required, could you perform 10 push-ups (either traditional or knee)?
- If required, could you run 1km?
- If required, could you perform 15 sit-ups?
- If required, could you perform 20 squats?
- Will you be able to perform today’s tasks over exertion?
- Do you feel capable of performing today’s tasks?
- Do you have any injuries currently that may affect your participation in today’s tasks?
- Do you have any pre-existing injuries that may impact today’s tasks?
- Do you feel you can perform today’s tasks in a safe manner?
- Do you feel any of today’s tasks could impact your physical health or can cause you any kind of injuries?

**Method for establishing the exercise protocol of the manuscript**

Targeted subjects were mainly from students and staff at Deakin University, Australia. Thus, the common age group was in the range of 20-40 years. A pilot study was conducted by selecting five subjects from each of the age groups 20-30 years and 30-40 years.

An estimate of a person's maximum heart rate can be calculated as 220 bpm minus their age. The moderate and high intensity zones were set using the below settings:

- Moderate physical activity 50-70% of the maximum HR
- High intensity physical activity 70-80% of the maximum HR

For the first phase with no inclination of the treadmill, we started the treadmill at a low speed setting and kept increasing the speed gradually every 5 minutes until the moderate physical activity HR, mentioned above, was achieved for the participant. The speeds at which the HRs were achieved were noted down, and the average was calculated and rounded to 5.5 km/h. Two lower speeds were then selected to achieve a variation of the HR.

For the second phase of the treadmill, we targeted a high intensity physical activity by setting the inclination of the treadmill to its maximum, 6.1^0^, as measured with a bevel gauge. The same procedure mentioned above was followed, however this time to achieve the maximum HR for the high intensity physical activity.

Similarly, two speeds of the spin cycle were selected to achieve moderate physical activity and high intensity physical activity HRs for each participant. The average of the speeds across five participants were calculated and used as the protocols’ set speeds.
